# New York State Inebriate Asylum

**Published:** 1858-10

**Authors:** 


OMISSION.—At the bottom of page 532, the words "to be continued" ought to have been inserted.

